# Non ABL-directed inhibitors as alternative treatment strategies for chronic myeloid leukemia

**DOI:** 10.1186/s12943-018-0805-1

**Published:** 2018-02-19

**Authors:** Michele Massimino, Stefania Stella, Elena Tirrò, Chiara Romano, Maria Stella Pennisi, Adriana Puma, Livia Manzella, Antonino Zanghì, Fabio Stagno, Francesco Di Raimondo, Paolo Vigneri

**Affiliations:** 1 Department of Clinical and Experimental Medicine, University of Catania, Via Santa Sofia, 78, Catania, 95123 Italy; 2 Center of Experimental Oncology and Hematology, A.O.U. Policlinico Vittorio Emanuele, Via Santa Sofia, 78, 95123 Catania, Italy; 3 Department of Surgical Medical Sciences and Advanced Technologies, University of Catania, Via Santa Sofia, 78, Catania, 95123 Italy; 4 Division of Hematology and Bone Marrow Transplant, University of Catania, Via Santa Sofia, 78, Catania, 95123 Italy; 5 Department of Surgery, Medical and Surgical Specialties, University of Catania, Via Santa Sofia, 78, Catania, 95123 Italy

**Keywords:** CML, BCR-ABL1, Therapeutic strategies, Immunological approaches

## Abstract

The introduction of ABL Tyrosine Kinase Inhibitors (TKIs) has significantly improved the outcome of Chronic Myeloid Leukemia (CML) patients that, in large part, achieve satisfactory hematological, cytogenetic and molecular remissions. However, approximately 15–20% fail to obtain optimal responses according to the current European Leukemia Network recommendation because of drug intolerance or resistance.

Moreover, a plethora of evidence suggests that Leukemic Stem Cells (LSCs) show BCR-ABL1-independent survival. Hence, they are unresponsive to TKIs, leading to disease relapse if pharmacological treatment is discontinued.

All together, these biological events generate a subpopulation of CML patients in need of alternative therapeutic strategies to overcome TKI resistance or to eradicate LSCs in order to allow cure of the disease.

In this review we update the role of “*non ABL-directed inhibitors*” targeting signaling pathways downstream of the BCR-ABL1 oncoprotein and describe immunological approaches activating specific T cell responses against CML cells.

## Background

Chronic Myeloid Leukemia (CML) is a myeloproliferative disorder characterized by neoplastic transformation of the Hematopoietic Stem Cell (HSC) which displays a cytogenetic marker derived from a reciprocal t9;22 translocation [1]. The ensuing Philadelphia (Ph) chromosome leads to the formation of the *BCR-ABL1* fusion oncogene encoding for a multi-domain BCR-ABL1 oncoprotein [2, 3]. BCR-ABL1 oncoprotein is the molecular hallmark of CML displaying constitutive tyrosine kinase activity that induces the activation of several intracellular pathways such as phosphoinositide 3-kinase (PI3K)/murine thymoma viral oncogene homolog (AKT)/mammalian target of rapamycin (mTOR), Rat Sarcoma proto-oncogene (RAS)/extracellular signal-regulated kinase (ERK) and Janus Kinases (JAK)/Signal Transducer and Activators of Transcription (STATs). Furthermore, BCR-ABL1-dependent improper signaling inhibits apoptosis and increases the proliferation rate of leukemic cells [4–7].

In 2001 the introduction of Imatinib Mesylate (IM), a semi-specific BCR-ABL1 tyrosine kinase inhibitor, improved the outcome of CML patients in chronic phase, generating unprecedented rates of hematologic, cytogenetic and molecular response [8–10]. Indeed, patients receiving IM 400 mg/daily in the IRIS (International Randomized Study of Interferon and STI571) study, achieved 83.3% 10-years survival [11]. Despite these excellent results, approximately 15–20% CML patients fail to achieve an optimal response as defined by the current European Leukemia Net (ELN) recommendations [11–14].

Several biological mechanisms responsible for IM failure have been described including BCR-ABL1-dependent and –independent mechanisms.

The former include: *i)* mutations in the ABL kinase domain which prevent TKI binding [15]; *ii)* amplification of the BCR-ABL1 oncogene [16, 17]; *iii)* high expression levels of the BCR-ABL1 mRNA [18].

The latter comprise: *i*) up-regulation of drug efflux pumps [19]; *ii*) down regulation of drug influx transporters [20]; *iii*) Lyn overexpression (Src-family kinase protein) [21] and *iv*) other BCR-ABL1-independent mechanisms [22].

To overcome IM-resistance, more potent second-generation (2G i.e. Dasatinib - DAS, Nilotinib - NIL, Bosutinib - BOS) and third-generation (3G i.e. Ponatinib - PON) TKIs have been developed and approved for the treatment of the disease [23–26].

However, while 2G and 3G TKIs present higher BCR-ABL1 inhibitory activity if compared to IM, they have failed to generate meaningful survival advantages for CML patients [27–30]. Moreover, it is now apparent that, despite complete inhibition of BCR-ABL1 kinase activity, TKIs are unable to eliminate quiescent Leukemic Stem Cells (LSCs) [4, 31, 32], as these cells are not “oncogene addicted” and therefore require alternative treatment strategies [32, 33].

In this review, we provide an update on the current knowledge of non ABL-directed inhibitors and immunological-targeting approaches as treatment strategies for CML patients achieving unsatisfactory responses to TKIs. In detail, we will focus on findings generated in primary CML cells, CML murine models and clinical trials.

### Farnesyl transferase inhibitors

Farnesyl Transferase Inhibitors (FT-Is) inhibit farnesyl transferase activity preventing isoprenoid-group transfer on different protein targets [34, 35]. Isoprenoid-group trasferring is a post-transcriptional modification that causes membrane migration of different proteins, such as RAS, resulting in their activation [36]. Activated RAS migrates in cellular membranes forming RAS-GTP which actives ERK- and AKT- dependent signaling modulating cell cycle progression, survival and proliferation. Improper RAS activation is common in several cancer types including CML [37], and different FT-Is were developed as anti-neoplastic drugs [34, 38, 39].

**Fig. 1:**
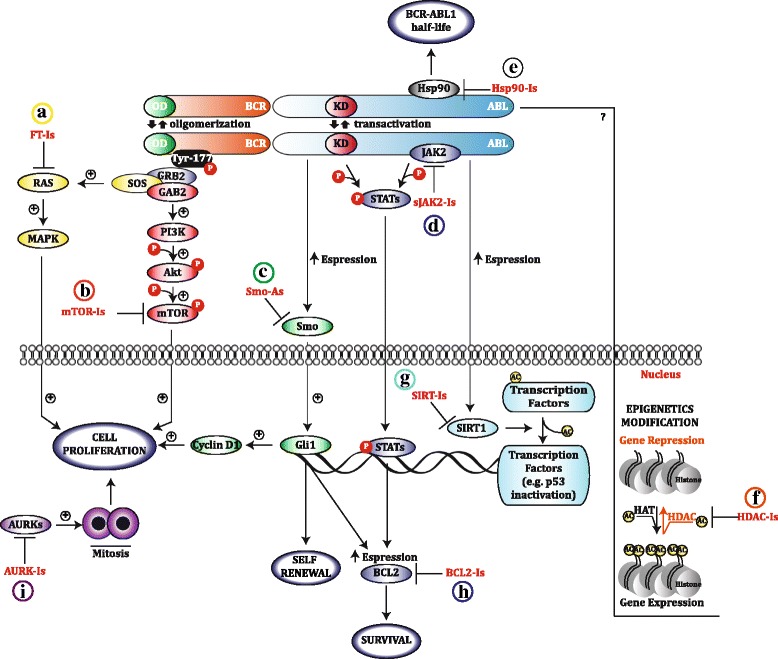
Schematic representation of the BCR-ABL1 signaling pathways targeted by non ABL-directed inhibitors. FT-Is (**a**) and mTOR-Is (**b**) inhibit RAS and mTOR activation resulting in cell proliferation arrest; Smo-As (**c**) inhibit the Hedgeohg signaling and reduce self-renewal, survival and cell proliferation; JAK-Is (**d**) suppress JAK2/STATs pathway reducing cell survival; Hsp-90-Is (**e**) reduce BCR-ABL1 half-life inducing its degradation; HDAC-Is (**f**) modify the histone acetylation state regulating gene expression; Sirt-Is (**g**) suppress the deacetylation activity of SIRT1; BCL2-Is (**h**) block the pro-survival activity of BCL-2 family members increasing apoptosis; AURK-Is (**i**) block the mitotic process by inhibiting of AURKs

In CML, constitutive RAS activation is promoted by BCR-ABL1 interaction with Grb2 (Growth factor receptor bound protein), SOS (Son Of Sevenless) and Gab2 (Grb2-associated binder 2) and plays a critical role in leukemogenesis [2, 40] (Fig. 1a). Tipifarnib (R115777) and Lonafarnib (SCH66336) are two potent and selective FT-Is with potential antileukemic activity in CML patients [41].

#### Tipifarnib (R115777)

Clinical data obtained from twenty-two CML patients with chronic or advanced disease that had failed Interferon-alpha (INFα) treatment demonstrated that Tipifarnib, as a single agent, induced complete or partial hematological responses and transient minor cytogenetic responses with a median duration of only 9 weeks [42]. In Phase I trials (NCT00040105), CML patients that had failed IM (50% with ABL kinase domain mutations), were treated with Tipifarnib in combination with IM. Co-treatment showed hematological and cytogenetic responses in 76% and 36% of patients, respectively. Moreover, four patients in cytogenetic remission (CyR) presented a BCR-ABL1 mutation (*n* = 1 T315I, *n* = 2 M244V, *n* = 1 E255K) [43].

#### Lonafarnib (SCH66336)

A pilot study investigated Lonafarnib efficacy in CML patients resistant or intolerant to IM. Only two of thirteen enrolled subjects showed hematological responses [44]. However, Lonafarnib administrated at different doses, showed greater efficacy when used in combination with IM. In particular, a Phase I study (NCT00047502) recruited CML patients who had failed IM observing hematological and cytogenetic responses in 35% of patients [45].

In summary, these data demonstrate that FT-I monotherapy showed little benefit for CML patients. However, their combination with IM may prove useful for CML subjects unresponsive to IM.

### mTOR inhibitors

mTOR Inhibitors (mTOR-Is) target the mammalian Target of Rapamycin (mTOR) [46], a serine/threonine kinase regulating cellular proliferation and metabolism [47] (Fig. 1b). Constitutive mTOR activation has been observed in different leukemia types, including CML [48]. BCR-ABL1 induces the PI3K/AKT pathway that results in mTOR phosphorylation, favoring tumor transformation [2, 48]. Several manuscripts have demonstrated the efficacy of mTOR-Is on primary and immortalized BCR-ABL-positive cell lines alone or in combination with TKIs [46, 49, 50].

#### Rapamycin (Sirolimus)

Rapamycin induces mTOR dephosphorylation resulting in reduced CML cell viability [51] and increased IM efficacy in resistant cells [52, 53]. To date, only one clinical trial is underway to evaluate the therapeutic potentials of Rapamycin in combination with DNA damaging agents such as Cytarabine or Etoposide in the accelerated or blast phase of CML (NCT00776373).

#### Everolimus (RAD001)

Everolimus blocks mTOR constitutive activation, reducing CML proliferation and increasing IM sensitivity [54, 55]. Interestingly, unlike Rapamycin, Everolimus can overcome IM resistance in BCR-ABL-positive quiescent cells transplanted in mouse recipients [56].

Everolimus therapeutic efficacy in CML patients, both alone and in combination with IM, is being evaluated in different clinical trials (NCT00081874), (NCT00093639).

#### BEZ235

BEZ235 is a dual PI3K-mTOR inhibitor tested on BCR-ABL1-positive cell lines. Published data demonstrated that the combination of BEZ235 and NIL induces apoptosis, inhibits tumor growth in CML xenograft models and impairs NIL resistance [57, 58]. A Phase I dose-finding study is being in patients with relapsed or refractory acute leukemia and advanced CML (NCT01756118).

#### Temsirolimus

To date, Temsirolimus is being investigated in a clinical trial in combination with IM (NCT00101088).

Even if the mTOR-Is have been thoroughly investigated in primary CML cells and in CML murine models recipient showing the ability to kill LSCs, to date, no data on CML patients are available, hence their therapeutic efficacy remains to be established.

### SMO antagonists

Smo Antagonists (Smo-As) inhibit Smoothend (Smo), a putative seven-transmembrane domain receptor which is a component of the Hedgeohg (Hh) pathway involved in a broad number of cellular mechanisms such as stem cell renewal, cell proliferation and survival (Fig. 1c). Binding of Hh human ligands, (Sonic Hedgeohg SHh, Desert Hedgeohg DHh, Indian Hedgeohg IHh) with Ptch (seven-transmembrane domain receptor Patched) causes a conformational change of Smo that actives the Glioma-associated oncogene (Gli1) transcription factor leading to faster cellular division and reduced apoptosis [59]. Hence, deregulation of the Hh pathway plays a critical role in the tumorigenesis and cancer progression [60].

CML patients showed higher Hh expression compared to healthy donors and IM treatment did not reduce these mRNA levels, suggesting that Hh over-expression was not dependent on BCR-ABL1 kinase activity [59, 61].

Dierks et al. reported that Smo up-regulation improves expansion of BCR-ABL1-positive LSCs [62]. Moreover, in-vivo experimental models using CML CD34-positive cells demonstrated that Hh inhibition in *Smo* knock-out mice, compromised both leukemic stem cell renewal and propagation [63]. Hence, this pathway represents a potential therapeutic target in BCR-ABL1-positive cells.

Smo-As have been investigated in ex-vivo studies as well as in several clinical trials.

#### LDE225 (Sonidegib/Erismodegib/Odomzo)

LDE225 significantly reduced colony forming ability and re-plating efficiency of CML CD34-positive cells and also decreases their Long Term Culture - Initiating Cell (LTC-IC) frequency. Furthermore, the combination of LDE225 with NIL reduced the engraftment of CML CD45-positive cells in NSG (NOD scid gamma) mice. [64]. At the present time, the LDE225-NIL combination is under investigation in a clinical trial enrolling patients that have failed at least one TKI (NCT01456676).

#### BMS833923 (XL139)

Two clinical trials have evaluated the efficacy of BMS833923 in CML. In the first study (NCT01218477) CML and Ph + Acute Lymphoblastic Leukemia (ALL) patients resistant to IM or NIL were exposed to the combination of BMS833923 and DAS. Only 1 of 27 patients in chronic phase attained a complete cytogenetic response while no patients with Ph + ALL or advanced CML displayed any clinical benefit [65]. In the second study (NCT01357655), newly diagnosed CP-CML patients were enrolled but no participants received the BMS8333923-DAS combination, as no recommended dose of the Smo-A drug could be found.

#### PF-04449913 (Gasdegib)

In preclinical studies, PF-04449913 impaired the multi drug resistance (MDR) mechanism in LSCs by down-regulating the *BCL2* (B-Cell Lymphoma 2) and/or *ABCA2* (ATP-Binding Cassette sub-family A member 2) oncogenes [66]. Furthermore, in CML xenograft models, treatment with PF-04449913 reduced the expansion of the leukemic stem cell suggesting a potential role for this compound in CML [67]. A Phase I dose escalation protocol (NCT00953758) investigated PF-04449913 safety in patients with different mieloproliferative disorders including CML, finding good tolerability at a dose which reduced Gli1 expression by Taqman array cards [68]. However, additional investigations are needed before this molecule can be considered for further development.

In conclusion, data obtained by ex-vivo studies or in mouse models suggest that inhibition of the Hh pathway interferes with both self-renewal and propagation of pluripotent BCR-ABL1-positive hematopoietic cells. Unfortunately, the unsatisfactory results obtained in CML patients currently preclude any significant role for these drugs in CML treatment.

### JAK2 inhibitors

JAK2 inhibitors (JAK2-Is) suppress JAK2 catalytic activity that modulates STATs transcription factors regulating the expression of genes involved in cell proliferation, differentiation and apoptosis (Fig. 1d). Published data report that JAK2 interacts with the ABL C-terminal leading to its constitutive activation [69]. Neviani and colleagues have demonstrated that, BCR-ABL1 induces constitutive JAK2 activation in quiescent leukemic cells in a kinase independent manner, reducing the activity of the Protein Phosphatase 2A (PP2A) tumor suppressor. Furthermore, PP2A reactivation by the small molecule FTY720, reduced JAK2 activation impairing stem cell self-renewal and overcoming TKI resistance [70].

JAK2 inhibitors (JAK2-Is) have also been combined with IM, NIL and DAS killing CML cells and restoring TKI-sensitivity in resistant CML cell lines [71–73].

#### Ruxolitinib

Using a combination of Ruxolitinib with NIL, Gallipoli and colleagues observed an increased apoptotic rate in CML cell lines and a reduction of the leukemic engraftment in CML murine models [74]. These data were supported by a Phase I study where CML patients exposed to Ruxolitinb and NIL achieved ≥1-log reduction in *BCR-ABL1* mRNA levels [75]. Several clinical trials are presently ongoing with Ruxolitinb alone or in combination with different TKIs in patients with advanced or resistant disease (NCT01702064), (NCT02253277), (NCT01751425), (NCT01914484), (NCT02973711).

#### BMS-911543

BMS-911543 displays cytotoxic effects in CML cell lines when administrated in combination with TKIs. Specifically, the exposure of BCR-ABL1-positive CD34 cells to BMS-911543 and DAS, eliminates TKI-insensitive leukemic stem cells, suggesting that the dual targeting strategy involving inhibition of both BCR-ABL1 and JAK2 may reduce the risk of developing TKI resistance in CML patients [76].

In conclusion, JAK2-Is combined with TKIs may represent a useful therapeutic approach for patients with advanced or resistant CML and may also contribute to the eradication of LSCs.

### Hsp90 inhibitors

Heat shock protein 90 (Hsp90) is a member of the Hsp family that encompass several ATP-dependent molecular chaperones constitutively expressed or induced by stress conditions such as hypoxia or toxin exposure (proteotoxic stress). They act preserving the correct folding of their client proteins and blocking their proteosomal degradation. Hsp90 shows high intratumoral expression and represents a poor prognostic indicator in cancer patients. Hsp90 inhibitors (Hsp90-Is) represent compounds of great interest as potential anti-leukemic agents [77–79].

Since, high Hsp90 expression inhibits BCR-ABL1 degradation, Hsp90-Is reduce BCR-ABL1 half-life (Fig. 1e) limiting the expansion of the leukemic clone [78]. The efficacy of four different Hsp90-Is has been evaluated in CML.

#### 17-AAG (Tanespimycin)

In preclinical experiments, 17-allylamino-17-demethoxygeldanamycin (17-AAG) showed low efficacy when used as monotherapy but increased apoptotic rates when administrated in combination with Histone Deacetylase Inhibitors (HDAC-Is) or IM [80, 81]. Two Phase I CML clinical trials evaluated 17-AAG alone (NCT00093821) or in combination with cytarabine (NCT00098423).

#### STA-9090 (Ganetespib)

Using in-vitro CML experimental models, Ying et al., compared the anticancer properties of STA-9090 and 17-AAG. STA-9090 was more potent than 17-AAG in reducing the proliferation of CML cells, suggesting that it may be a useful agent for CML patients [82]. Both Phase I and Phase II trials are being STA-9090 efficacy in CML patients with advanced (NCT00964873) or relapsed (NCT00858572) disease.

#### BIIB021

BIIB021 reduces BCR-ABL1 protein expression thereby inducing significant growth inhibition in CML cell lines both sensitive and resistant to TKIs. In addition, BIIB021 also triggers autophagy by repressing the AKT-mTOR pathway and thus reactivating autophagy-inducer Ulk1 (unc-51 like autophagy activating kinase 1) [83].

#### Novobiocin

Novobiocin is a potent inhibitor of CML cell proliferation, with weak effects on CD34-positive cells derived from healthy donors. Furthermore, co-treatment of Novobiocin with IM reduced the proliferation of TKI-resistant cells, suggesting that this combination may be useful to overcome the mechanisms leading to IM failure [84].

In summary, Hsp90-Is generated promising results against primary and immortalized CML cells and in CML mouse models. However, the lack of data in CML patients requires further studies to asses the effectiveness of Hsp90-Is for CML treatment.

### Histone Deacetilase and Sirtuin inhibitors

*Histone Deacetilase Inhibitors (HDAC-Is)* are small-molecules that block HDAC enzymes involved in epigenetic modifications that regulate histone acetylation state. In general, while histone acetylation carried by Histone Acetyl Transferases (HATs) determines a chromatin permissive state that favors gene expression, histone deacetylation performed by HDACs, overturn this biological event inducing gene repression [85] (Fig. 1f).

Different HDAC isoforms, belonging to three different classes, are overexpressed in several cancer types. This up-regulation is associated with a reduction in both overall and disease-free survival suggesting a possible role for HDAC-Is as antitumor drugs [86]. Although no data support the involvement of HDAC in BCR-ABL1-dependent transformation, many authors and several clinical trials have evaluated HDAC-Is activity in CML.

#### SB939 (Pracinostat)

One of the biological mechanisms responsible for TKI resistance, is the intronic deletion polymorphism of the *BIM* gene. SB939 restores IM sensitivity in CML CD34-positive cells displaying the intronic deletion polymorphism of the *BIM* gene by repairing its pre-mRNA splicing, suggesting that patients presenting this polymorphism, may benefit from the combination of SB939 and IM [87]. Okabe et al. have associated two different HDAC-Is with Tozasertib (Aurora Kinase Inhibitor) in both immortalized and primary CML cells. They found that the synergic effect of SB939 or Vorinostat in combination with Tozasertib results in an increased apoptotic rate [88].

#### Vorinostat

Several manuscripts have found that the combination of Vorinostat with aurora kinase inhibitors (AURK-Is) or TKIs kills primary CML cells, Baf3 cells expressing different BCR-ABL1 mutants and also shows antileukemic properties in CML mouse models. [88, 89]. These data are also supported by CML clinical trials of Vorinostat in combination with the DNA damaging agent decitabine (NCT00275080), DAS (NCT00816283) or with the cyclin-dependent kinase inhibitor flavopiridol (NCT00278330).

#### LBH589 (Panobinostat)

LBH589 is an HDAC-I with potent antiproliferative activity in several cancer cell lines [90]. LBH589 inhibits Hsp90 promoting the proteosomal degradation of Hsp90 client proteins such as BCR-ABL1. Zaritskey et al. have investigated the therapeutic efficacy of this drug in a Phase II study (NCT00451035) including CML patients resistant to at least two previous TKIs. Of the twenty-nine recruited CML patients, only one showed a hematological remission with eradication of a T315I-positive clone in the absence of any CyR [91]. LBH589 has also been extensively studied as a potential anti-leukemic drug in combination with different TKIs. Matsuda and colleagues reported that LBH589 increased PON cytotoxicity in IM-resistant CML cell lines [92]. LBH589 is also being evaluated in combination with IM in CML patients in CyR with residual disease detectable by Q-PCR (NCT00686218).

These results suggest that HDAC-Is have questionable efficacy as single agents while they may be promising therapeutic agents when administrated in combination with additional anti-cancer drugs in patients failing TKIs.

*Sirtuin Inhibitors (Sirt-Is)* are a broad range of pharmaceutical agents inhibiting class III HDAC enzymes called Sirtuins (SIRTs) (Fig. 1g). These proteins play a key role in both healthy and cancer cells by mediating changes in the activation of oxidative stress. In mammals, seven SIRTs (SIRT1–SIRT7) have been identified which display a conserved core NAD^+^-binding domain and exhibit deacetylation and ADP-ribosylation activities [93]. Among all sirtuins, SIRT1 has been investigated in different hematological malignances including CML [94]. SIRT1 is overexpressed in primary and immortalized CML cells and a *SIRT1* knock-out represses BCR-ABL1 transforming activity in mice recipients [95, 96]. Sirt-Is such as tenovin-6, sirtinol and nicotinamide have been investigated in CML experimental models.

#### Tenovin-6 (TV-6)

Is a small-molecule that inhibits SIRT1 and SIRT2 resulting in p53 acetylation and activation [94]. The combined pharmacological inhibition of SIRT1 (by TV-6) and BCR-ABL1 (by IM) decreases cell proliferation, promotes apoptosis of CML progenitors and impairs CML engraftment in immunodeficient mice [95].

#### Sirtinol

Unlike TV-6, sirtinol is a SIRT1 specific inhibitor with anti-cancer properties in different tumors [97]. Wang et al. reported that SIRT1 overexpression promotes the acquisition of genetic mutations that, in turn, cause TKI resistance. Exposure to Sirtinol overcomes resistance to IM, NIL and DAS. [98].

In conclusion, the ability of Sirt-Is to maintain genomic stability and to reduce the LSCs pool, makes these compounds promising tools for CML treatment.

### BCL2 inhibitors

Studies of gene and protein expression have shown that alternative splicing of multiple BCL2 family members facilitate the expansion of quiescent CML stem cells [99, 100] and reduce their apoptotic rate [101].

As BCL2 inhibitors (BCL2-Is) overturn these biological effects (Fig. 1h), they have been considered for the treatment of CML.

#### Sabutoclax

Sabutoclax, a pan-BCL2 inhibitor, sensitizes LSCs in the bone marrow niche to TKIs. A recent study has shown that exposure of CML CD34-positive cells to Sabutoclax increases DAS efficacy reducing engraftment of LSCs in mice [102].

#### Obatoclax

Preclinical evidence suggests that the pan-BCL2 inhibitor Obatoclax reduces colony formation in Ph + CD34-positive progenitors [103]. A Phase I study has been designed to evaluate the safety of Obatoclax in different myleoproliferative disorders, including CML (NCT00438178).

#### Venetoclax (ABT-199)

Unlike Sabutoclax and Obatoclax, Venetoclax displays BCL2-selective antagonism with modest activity against CML progenitors when used as single agent. However, Ko and colleagues have recently shown that Venetoclax enhances IM cytotoxicity on CML progenitors [104].

In conclusion, although BCL2 inhibition may become a useful strategy in the future, the lack of clinical data in CML patients currently excludes this class of drugs from CML therapy.

### Aurora kinase inhibitors

Aurora kinase inhibitors (AURK-Is) suppress the serine-threonine kinase activity of the AURK family that regulates cell division [105–107] (Fig. 1i). Three isoforms of the Aurora Kinases (AURORA-A -B and -C) modulate chromosome condensation and orientation playing a critical role in the control of the mitotic machinery. Hence, dysregulation of their activity generates chromosomal abnormalities driving DNA alterations responsible for cell transformation [107]. On the basis of these considerations, the AURKs have been considered potential therapeutic targets for the development of anticancer drugs [106]. Although, to date, the BCR-ABL1/AURK correlation with CML progression is unclear, the role of AURK-Is in CML treatment has been exstensively investigated [105].

#### MK-0457 (VX-680 or Tozasertib)

MK-0457 is active against immortalized CML cell lines and has also shown the ability to revert advanced CML patients expressing the T315I mutant to the chronic phase of the disease [108, 109]. These promising data have resulted in the design of a Phase II study (NCT00405054) that showed cytogenetic and hematologic responses in advanced CML patients [110]. Finally, a Phase I dose escalation study of MK-0457 in combination with DAS is also ongoing (NCT00500006).

#### PHA-739358 (Danusertib)

Unlike MK-0457, PHA-739358 is a dual inhibitor of AURK and ABL (wild-type and mutated, including T315I), which showed promising activity both in leukemia and solid tumors. In detail, Danusertib exerts growth inhibition in immortalized BCR-ABL1-positive cells and in CML CD34-positive progenitors derived from patients sensitive or resistant to TKIs [111, 112]. In a Phase I study, used as a single agent, PHA-739358 displayed acceptable toxicity and induced hematologic and cytogenetic responses in patients with advanced CML expressing the T315I mutant [113].

#### AKI603

AKI603 is an aurora kinase A inhibitor that exerts its antiproliferative activity by arresting CML cells sensitive or resistant to IM in the G2/M phase of the cell cycle. [114]. AKI603 also abrogates the growth of xenografted BCR-ABL1 T315I mutant cells in nude mice and restore IM ability to reduce their colony forming potential [115].

#### MLN8237 (alisertib)

Like AKI603, MLN8237 is an Aurora Kinase A inhibitor but it induces CML cell death by decreasing expression of Apollon, a protein that modulates cell division and apoptosis. In-vitro CML experimental models showed that MLN8273 induces apoptosis in cells expressing both wt and mutant BCR-ABL1. Moreover, MLN8273 improves NIL activity increasing CML CD34-positive cell death and reducing tumor growth in recipient mice [116].

#### AT9283

This multitarget kinase inhibitor, shows activity against CML cell lines and is able to reduce the engraftment of primary BCR-ABL1-positive cells [117]. A Phase I/II study is being the efficacy safety of AT9283 in patients with refractory hematological malignancies including CML (NCT00522990).

All together, these data indicate a likely role for AURK-Is as a useful therapeutic resource for patients with advanced CML resistant to TKIs.

### Protein translation inhibitor - Omacetaxine

**Fig. 2:**
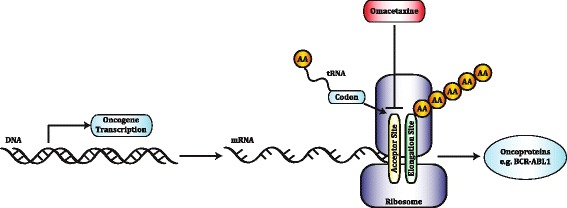
Schematic representation of the mechanism of action of Omacetaxine. Oncogene transcription leads to mRNA translation that induce oncoprotein synthesis. Omacetaxine reduces BCR-ABL1 expression levels by blocking the ribosome t-RNA aminoacil acceptor site that results in a protein elongation arrest

Omacetaxine binds the ribosome aminoacyl-tRNA acceptor site, thereby inhibiting the synthesis of different oncoproteins including BCR-ABL1 [118] (Fig. 2). Experimental data on primary BCR-ABL1-positive cells [119] and different clinical trials have demonstrated the efficacy of Omacetaxine as a therapeutic agent in CML.

Cortes and colleagues used Omacetaxine in CML patients resistant or intolerant to TKIs and obtained meaningful hematological and cytogenetic remissions [120, 121]. Furthermore, the same data were obtained in a Phase II study, (NCT00375219), enrolling patients with the T315I mutation [122].

Following these clinical data, the FDA approved Omacetaxine for the treatment of CML patients that do not benefit from TKIs with specific attention to patients carrying the T315I substitution.

Clinical studies and results from non ABL-directed Inhibitors are summarized in Table 1.

**Table 1:** Clinical studies and results from non ABL-directed Inhibitors

Non ABL-directed Inhibitors	Clinical Study	Drugs Combination	Patient Characteristics (pts)	Results
FT-Is				
*Tipifarnib*	(42)	–	CP, AP, BP (*n* = 22)	HR: 27% CP, 4% AP CyR: 18% CP
	PhaseI NCT00040105^(43)^	IM	CP having ABL KD mutation (*n* = 25)	HR: 76% CyR: 36%
*Lonafarnib*	Pilot Study^(44)^	–	CP, AP IM resistant (*n* = 13)	HR: 8% CP, 8% AP
	PhaseI NCT00047502^(45)^	IM	CP, AP, BP (*n* = 23)	HR: 9% CP, 17% AP/BP CyR: 4% CP, 4% AP/BP
mTOR-Is				
*Rapamycin*	PhaseI/II NCT00776373	Cytarabine Etoposide	AP, BP	NDP
*Everolimus*	PhaseI/II NCT00081874	–	BP	NDP
	PhaseI/II NCT00093639	IM	CP	NDP
*BEZ235*	PhaseI NCT01756118	–	AP, BP	NDP
*Temsirolimus*	PhaseI NCT00101088	IM	AP, BP	NDP
Smo-As				
*LDE225*	PhaseI NCT01456676	NIL	AP, BP	NDP
*BMS833923*	PhaseI NCT1218477^(66)^	DAS	CP, AP, BP (*n* = 27)	CyR: 4% CP PA/BP: no responded
	PhaseI NCT01357655	DAS	CP	No participants were enrolled
*PF-04449913*	Phase I NCT00953758	–	CP	Safety, Pharmacokinetics and Pharmacodynamics study
JAK2-Is				
*Ruxolitinib*	PhaseI NCT01702064	NIL	CP	ongoing
	PhaseI/II NCT02253277	NIL	CP, Ph + ALL	ongoing
	PhaseI/II NCT01751425	–	CP with MRD	ongoing
	PhaseI/II NCT01914484	NIL	AP, BP, Ph + ALL	ongoing
	PhaseI/II NCT02973711	NIL	CP	ongoing
Hsp90-Is				
*17-AAG*	PhaseI NCT00093821	–	BP	NDP
	PhaseI NCT00098423	Cytarabine	BP	NDP
*STA-9090*	PhaseI NCT00964873	–	BP	NDP
	PhaseI NCT00858572	–	refractory or relapsed CML	NDP
HDAC-Is				
*Vorinostat*	PhaseI NCT00275080	Decitabine	BP	NDP
	PhaseI NCT00816283	DAS	AP, BP	NDP
	PhaseI NCT00278330	Flavopiridol	BP	NDP
*LBH589*	Phase II/III NCT00451035^(91)^	–	CML TKIs resistant (*n* = 29)	HR: 3%
	PhaseI NCT00686218	IM	CP with MRD	NDP
BCL2-Is				
*Obatoclax*	PhaseI NCT00438178	–	BP	NDP
AURK-Is				
*MK-0457*	Phase I/II^(108)^	–	AP, BP, Ph + ALL All with T315I (*n* = 18)	HR: 39% AP/BP CyR: 5% Ph + ALL
	PhaseII NCT00405054^(110)^	–	AP, BP, Ph + ALL All with T315I (*n* = 52)	CyR: 8% CP, 6% AP/BP HR: 4% CP
	PhaseI NCT00500006	DAS	CP	No Data Results Posted
*PHA-739358*	PhaseI^(113)^	–	AP, BP (n = 29)	HR: 7% AP/BP, 7% Ph + ALL CyR: 3% AP/BP, 3% Ph + ALL MR: 3% Ph + ALL
*AT9283*	PhaseI NCT00522990	–	CP, AP, BP	NDP
*XL288*	PhaseI NCT00464113	–	CP, AP, BP, Ph + ALL	NDP
PT-Is				
*Omacetaxine*	PhaseII^(120)^	–	CP, TKIs resistant (*n* = 46)	HR: 67%, CyR: 22
	PhaseI/II^(121)^	–	CP, previously exposed to TKIs (*n* = 81)	HR: 81%, CyR: 20%
	PhaseII NCT00375219^(122)^	–	CP, BCR-ABL1 T315I mutant (*n* = 62)	HR: 77%, CyR: 22%

*FT-Is* Farnesyl Transferase Inhibitors, *mTOR-Is* mammalian Target of Rapamycin, *Smo-As* Smo Antagonists, *JAK2-Is* JAK2 Inhibitors, *Hsp-90-Is* Hsp-90 Inhibitors, *HDAC-Is* HDAC Inhibitors, *BCL2-Is* BCL2 Inhibitors, *AURK-Is* Aurora Kinase Inhibitors, *PT-Is* Protein Translation Inhibitors, *HR* Hematological Remission, *CyR* Cytogenetic Remission, *CP* Chronic Phase, *AP* Accelerated Phase, *BP* Blast Phase, *NDP* No Data Posted, *MRD* Minimal Residual Disease

### Immunological approaches

The immune response against cancer is impaired by an immune escape of the tumor cells [123]. Over the past decade, different investigators have studied vaccines activity in CML patients using BCR-ABL1 as specific antigen. Leukemia Associated Antigens (LAAs) and Dendritic Cell Vaccines (DCs) have also been investigated with the aim of inducing a T cell immune response against BCR-ABL1-expressing cells [99]. Furthermore, use of the immune-checkpoint blockade (ICB) has also been assessed.

#### BCR-ABL1 as a specific antigen

Usually BCR-ABL1 immunogenic peptides are formed by an amino acid sequence of the e13a2 or e14a2 break-point region [124]. Different authors have investigated the efficacy of BCR-ABL1 immune-peptides in CML.

The EPIC (Evaluation of Peptide Immunisation in CML) study accrued nineteen patients that were vaccinated using e14a2 peptides. Thirteen patients, in cytogenetic remission after IM, showed late T cell immune response to BCR-ABL1 peptides and achieved a 1-log decrease in BCR-ABL1 transcripts [125].

Nitin and colleagues investigated the efficacy of a mixture of immune-peptides in ten CML patients expressing e13a2 or co-expressing e13a2/e14a2 BCR-ABL1 isoforms. Three patients achieved a 1-log reduction in BCR-ABL1 mRNA levels and 3 additional patients developed a major molecular response. However, these responses have not been stable over time, suggesting that this therapeutic approach may only transiently improve molecular response in CML patients [126].

In a Phase 2 trial (NCT00267085), patients previously exposed to IM and showing complete cytogenetic remission but not a major molecular response were subjected to vaccination using the CMLVAXB2 or CMLVAXB3 peptides against the e13a2 and e14a2 BCR-ABL1 isoforms, respectively. Three patients out of ten achieved a 1-log reduction in BCR-ABL1 mRNA levels.

An interim analysis of a Phase II Multicenter GIMEMA CML Working Party trial reported that CML patients with minimal residual disease during IM treatment obtained a reduction of their disease burden after being exposed to the peptide vaccine CMLVAX100 (derived from BCR-ABL1 e14a2 isoform plus molgramostin, a leucocyte growth factor and QS-21 as immunoadjuvant) [127]. Furthermore, Bocchia et al. demonstrated that the combination CMVAX100 with GMCSF induced 50% of *BCR-ABL1* mRNA levels reduction in patients previously exposed to IM and/or IFN [128].

The same group also described a patient that received a vaccine based on the e13a2 BCR-ABL1 isoform (CMLb2a2–25mer), achieving undetectable *BCR-ABL1* transcripts in both peripheral blood and bone marrow [129].

In summary, the vaccines against BCR-ABL1 break-points have shown the ability to reduce residual disease in TKI-treated patients achieving cytogenetic remission. Several clinical trials are being this therapeutic approach (NCT00428077), (NCT00466726), (NCT00004052).

#### Leukemia associated antigens (LAAs)

Leukemia Associated Antigens (LAAs) are overexpressed in multiple leukemias including CML. Different LAAs have been identified as potential targets for vaccine synthesis and CML therapy [124, 130]. Among them, the most promising are: *i*. the immunopeptide against the Wilms tumor oncogene (WT1), frequently overexpressed in CML patients. When this immunopeptide associated with IM, it may induce deep molecular response [131]. Currently, one clinical trial is evaluating the efficacy of this approach (NCT00004918); *ii.* K562/GM-CSF (GVAX), a cell-based vaccine derived from K562 cells genetically modified to produce granulocyte-macrophage colony-stimulating factor (GM-CSF) and a number of LAAs which recruit dendritic cells and activate T cell-mediated CML-specific immune responses. GAVX has been shown to reduce BCR-ABL1 transcript levels in CML patients [132].

Overall, the data generated in CML preclinical models and clinical report indicate a promising role for immune-dependent therapies for CML treatment.

#### DCs vaccine (dendritic cells)

DCs are antigen-presenting cells that induce humoral and cellular immune responses. In CML, progenitor cells drive the formation of both leukemic clones and DCs. Since 98% of them express the BCR-ABL1 oncoprotein, these cells represent a potential target for immunological therapy [124]. Previous published data indicates that CML-DCs present antigen-processing defects as a consequence of their reduced capacity to capture antigens if compared with normal DCs [133]. Furthermore, in two clinical trials, DCs injections did not generate any response [134, 135].

In conclusion, DCs-based vaccines appear unlikely to be of any meaningful value for CML treatment in the foreseeable future.

#### Immune-checkpoint blockade (ICB)

Cancer immunotherapy based on immune-checkpoint blockade (ICB) employs monoclonal antibodies against negative immune-regulator checkpoints such as cytotoxic T-lymphocyte antigen 4 (CTLA-4), programmed death 1 (PD-1) and its ligands (PD-L1, PD-L2) [136].

CML-specific cytotoxic T Lymphocytes (CTLs) show high PD-1 levels, whereas CML cells express PD-L1. In murine CML models, abrogation of PD-1 expression increases overall survival [137, 138] suggesting that blocking the PD-1/PD-L1 pathway may represent a new therapeutic strategy for CML.

Recently, Schutz demonstrated a correlation between CTLA-4-ligand CD86 expression and risk of disease relapse after TKI discontinuation. Indeed of 122 patients that had ceased TKIs, those expressing lower CD86 levels showed a 70% relapse-free survival suggesting that CD86 expression may be an early indicator of poor treatment-free remission probability [139].

A clinical trial (NCT01822509) is presently evaluating the efficacy of the combination ipilimumab (anti-CTLA-4) plus nivolumab (anti-PD-1) in patients with hematologic malignancies, including CML, relapsed after allogeneic hematopoietic cell transplantation.

Clinical studies and results from immune strategies are summarized in Table 2.

**Table 2:** Clinical studies and results from immune strategies

Immune-peptide	Clinical Study	Drug Combinations	Patient Characteristics (pts)	Results (pts%)
BCR-ABL1 as specific antigen				
*e14a2*	PhaseI/II^(125)^	IM	CP in CyR (*n* = 19)	(68%) < 1-log BA mRNA
*e13a2, e14a2*	PhaseII^(126)^	IM	CP in CyR (*n* = 10)	(30%) < 1-log BA mRNA (30%) MMR (transient response)
*CMLVAXB2 (e13a2) CMLVAXB3 (e14a2)*	PhaseII NCT00267085	IM	CP in CyR (n = 10)	(30%) ↓< 1-log BA mRNA
*CMLVAX100 (e14a2)*	PhaseII^(127)^	IM IFN	CP in SRD (all *n* = 16) IM (n = 10); IFN (n = 6)	IM: (50%) CyR and (30%) BA UD IFN: (83%) CyR
*CMLVAX100-GMCSF*	PhaseII^(128)^	IM IFN	CP in MRD (*n* = 43)	(51%) ↓50% BA mRNA
*CMLb2a2–25 (e13a2)*	Case Study^(129)^	–	CP in CyR	BA UD
*e13a2, e14a2*	PhaseII NCT00428077	–	MRD (n = 4)	(100%) < 1-log BA mRNA
*e13a2*	PhaseII NCT00466726	IM	CP in MRD	NDP
*e13a2*	PhaseII NCT00004052	–	CP in HR	NDP
LAAs				
*WT1*	Case Study^(131)^	IM	MRD	↓ BA mRNA
	PhaseI/II NCT00004918	–	CP	NDP
*GVAX*	(132)	IM	CyR (n = 19)	(68%) ↓ BA mRNA
ICB				
* Ipilimumab* * Nivolumab*	PhaseI NCT01822509	–	CP	ongoing

*LAAs* Leukemia Associated Antigens, *ICB* Immune-checkpoint blockade, *HR* Hematological Remission, *CyR* Cytogenetic Remission, *CP* Chronic Phase, *AP* Accelerated Phase, *BP* Blast Phase, *NDP* No Data Posted, *MRD* Minimal Residual Disease, *UD* Undetectable, *SRD* Stable Residual Disease

## Conclusion

TKIs that interfere with BCR-ABL1 signaling currently represent the first line and second line treatment of choice for most CML patients [26].

However, BCR-ABL1-dependent or –independent resistance as well as BCR-ABL1-independent LSCs survival, partially undermine TKIs efficacy. Hence, a subgroup of CML patients is clearly in need of alternative therapeutic approaches. In this review we focused our attention on a range of pharmacological agents -non ABL-directed inhibitors- against different targets involved in BCR-ABL1-dependent leukemic transformation.

We summarized data showing that FT-Is in combination with TKIs, Omacetaxine, AURK-Is and JAK2-Is have demonstrated efficacy in CML patients. We have also outlined clinical data demonstrating that vaccination against WT1 antigen, in combination with IM may represent a potential strategy to reduce *BCR-ABL1* mRNA levels or induce cytogenetic remissions. However, with the exception of Omacetaxine, none of the above indicated compounds have received approval for CML treatment. Furthermore, while there are several ongoing clinical trials evaluating the association of Ruxolitinib with NIL, at the current time it appears unlikely that other promising agents (i.e. FTY720, Hsp90-Is, AURK-Is and anti-WT1 antibodies) will undergo clinical development for the treatment of the disease.

The unsatisfactory results obtained with most of the non ABL-direct inhibitors has fostered additional research in the field that is currently investigating alternative strategies including: *i*) a WNT (homologus wingless)-targeting drug to modulate stem cell survival (PRI-724, clinical trial NCT01606579), *ii*) HDM2 (known as mdm-2, mouse double minute-2) inhibition to increase p53 half-life (RG7112, clinical trial NCT00623870), *iii*) a CXCR4 (CXC-chemokine receptor 4) antagonist as a hematopoiesis regulator (BL8040, clinical trial NCT02115672), *iv*) an ABL allosteric modulator (i.e. ABL001, clinical trial NCT02081378).

In summary, non ABL-directed inhibitors have often showed ability to overcome TKI resistance in primary CML cells or to eradicate the LSCs in mouse models. However, they displayed questionable efficacy in CML patients. Likewise, immunological approaches may be useful to improve molecular response, but this effect is often transient.

Finally, while the use of ICB may represent promising approaches to eradicate LSCs and predict molecular relapse of the disease after TKI discontinuation, these immune-based strategies seem far from achieving clinical relevance for CML therapy.
